# Supplementary material for: Trends in Prediabetes and Non-Alcoholic Fatty Liver Disease Associated with Abdominal Obesity among Korean Children and Adolescents: Based on the Korea National Health and Nutrition Examination Survey between 2009 and 2018
Source: Biomedicines. 2022 Mar 2;10(3):584. doi: 10.3390/biomedicines10030584 (PMC8945340; doi:10.3390/biomedicines10030584)
Supplement: Supplementary file 1 [file biomedicines-10-00584-s001.zip › biomedicines-1593517-supplementary.pdf]

SUPPLEMENTARY DATA

Trends in Prediabetes and Non-alcoholic Fatty Liver Disease associated with Abdominal Obesity among Korean Children and Adolescents: Based on the Korea National Health and Nutrition Examination Survey between 2009 and 2018

Kyungchul Song[1], Goeun Park[2], Hye Sun Lee[2], Myeongseob Lee[1], Hae In Lee[1], Jungmin Ahn[1,3], Eunbyoul Lee[1,4], Han Saem Choi[1], Junghwan Suh[1], Ahreum Kwon[1], Ho-Seong Kim[1], and Hyun Wook Chae[1,*]

[1]Department of Pediatrics, Severance Children's Hospital, Endocrine Research Institute, Yonsei University College of Medicine, Seoul, South Korea

[2]Biostatistics Collaboration Unit, Yonsei University College of Medicine, Seoul, South Korea

[3]Department of Pediatrics, Jeju National University, College of Medicine and Graduate, School of Medicine, Jeju, South Korea

[4]Department of Pediatrics, Catholic Kwandong University, International St.Mary's Hospital, Incheon, South Korea

*Corresponding author: Hyun Wook Chae, Department of Pediatrics, Severance Children's Hospital, Endocrine Research Institute, Yonsei University College of Medicine, Seoul, South Korea, 03722. Phone: +82-2-2019-3350; email: hopechae@yuhs.ac

Table S1. Trend of characteristics of participants according to abdominal obesity

Variable	2009	2010	2011	2012	2013	2014	2015	2016	2017	2018	*Total*	*P*	*P* for trend
Abdominal obesity	n=63	n=66	n=68	n=45	n=49	n=46	n=79	n=75	n=48	n=50	n=589		
Age, yr	14.18 (0.37)	13.98 (0.45)	14.56 (0.33)	14.55 (0.44)	14.61 (0.33)	14.90 (0.41)	14.42 (0.33)	14.74 (0.32)	15.33 (0.38)	14.86 (0.32)	14.63 (0.12)	0.481	0.015
Sex (male), %	43.21 (6.86)	39.77 (7.73)	47.07 (6.73)	51.87 (9.69)	55.57 (7.87)	45.01 (8.37)	49.22 (5.88)	48.39 (5.99)	48.92 (8.25)	60.30 (8.64)	48.94 (2.41)	0.844	0.169
Height SDS	1.00 (0.14)	0.60 (0.22)	0.56 (0.14)	0.85 (0.23)	0.57 (0.18)	0.74 (0.21)	0.66 (0.15)	0.71 (0.12)	0.35 (0.23)	0.87 (0.13)	0.68 (0.06)	0.294	0.428
Weight SDS	2.11 (0.14)	2.06 (0.09)	2.17 (0.12)	2.18 (0.13)	2.23 (0.18)	2.30 (0.14)	2.30 (0.13)	2.11 (0.08)	2.14 (0.22)	2.40 (0.12)	2.20 (0.04)	0.548	0.196
BMI SDS	2.04 (0.13)	2.17 (0.14)	2.32 (0.13)	2.19 (0.12)	2.37 (0.19)	2.37 (0.16)	2.45 (0.13)	2.15 (0.09)	2.37 (0.20)	2.41 (0.14)	2.29 (0.04)	0.386	0.076
BMI percentile												0.292	0.058
Normal, %	9.76 (4.82)	5.87 (2.84)	5.92 (3.03)	0.45 (0.45)	12.94 (5.93)	8.90 (4.94)	3.10 (1.80)	5.10 (2.30)	4.63 (4.33)	3.63 (2.74)	5.74 (1.10)		
Overweight, %	27.71 (6.70)	24.63 (6.74)	14.40 (5.29)	16.06 (6.56)	11.18 (4.27)	10.66 (4.62)	15.71 (4.17)	21.64 (5.03)	8.04 (3.89)	13.76 (6.02)	16.20 (1.71)		
Obesity, %	62.53 (7.15)	69.50 (6.99)	79.68 (5.71)	83.49 (6.65)	75.88 (7.03)	80.44 (6.51)	81.19 (4.46)	73.26 (5.58)	87.33 (5.37)	82.61 (6.14)	78.06 (1.93)		
Glucose, mg/dL	89.08 (1.12)	90.57 (1.53)	90.64 (0.94)	88.19 (1.29)	90.62 (1.19)	93.56 (0.92)	93.23 (1.41)	92.47 (1.02)	92.12 (0.96)	93.68 (0.89)	91.59 (0.38)	0.002	<0.01
Prediabetes, %	12.58 (5.15)	12.53 (4.85)	8.39 (3.71)	4.87 (2.77)	9.68 (4.29)	10.72 (4.67)	17.74 (4.99)	16.12 (4.86)	14.34 (5.42)	15.61 (5.12)	12.54 (1.53)	0.654	0.129
AST, IU/L	21.35 (1.84)	19.96 (1.00)	24.26 (1.79)	22.23 (3.27)	22.36 (1.86)	20.23 (1.58)	26.20 (3.99)	20.49 (1.17)	23.94 (1.89)	27.72 (2.51)	23.02 (0.78)	0.075	0.052
ALT, IU/L	27.28 (4.42)	21.75 (2.34)	35.87 (5.98)	26.00 (4.98)	29.48 (3.95)	24.08 (2.90)	33.44 (7.49)	25.91 (3.13)	33.63 (4.57)	43.55 (8.07)	30.42 (1.75)	0.072	0.059
NAFLD, %	31.91 (6.77)	20.59 (5.50)	42.69 (6.38)	33.48 (8.77)	44.52 (8.07)	32.37 (9.05)	36.53 (5.88)	34.93 (6.43)	51.67 (8.52)	57.79 (9.85)	38.80 (2.40)	0.060	<0.01
Prediabetes and NAFLD, %	4.19 (2.84)	4.44 (2.69)	1.38 (1.35)	1.62 (1.56)	4.36 (3.12)	4.65 (3.18)	3.78 (3.39)	3.22 (2.23)	5.91 (3.62)	11.20 (4.87)	4.32 (0.96)	0.555	0.117
Total cholesterol, mg/dL	166.17 (5.89)	165.57 (3.56)	170.42 (5.67)	172.31 (6.72)	165.79 (3.56)	170.24 (4.00)	169.64 (3.52)	168.98 (4.37)	179.74 (3.19)	177.32 (3.55)	170.72 (1.47)	0.062	0.025
HDL-C, mg/dL	44.00 (1.30)	44.79 (1.41)	43.75 (1.19)	46.93 (1.80)	47.60 (1.74)	47.21 (1.39)	46.88 (1.45)	47.09 (1.12)	44.43 (0.98)	44.48 (1.41)	45.75 (0.44)	0.224	0.444
LDL-C, mg/dL	100.49 (5.14)	102.33 (3.33)	108.12 (5.38)	103.20 (5.78)	97.90 (3.33)	102.06 (3.08)	106.77 (3.09)	102.22 (3.75)	112.58 (2.71)	110.30 (3.28)	104.94 (1.31)	0.030	0.049
Triglycerides, mg/dL	108.40 (6.80)	110.59 (9.98)	117.21 (8.75)	130.05 (11.79)	106.33 (8.11)	118.13 (11.24)	108.66 (7.15)	102.85 (6.22)	120.79 (9.42)	126.74 (9.96)	114.59 (2.89)	0.419	0.530
Energy, kcal/day	1885.34 (78.27)	2043.34 (123.19)	1982.00 (115.02)	1945.37 (144.72)	2013.13 (139.15)	2523.89 (259.07)	2180.56 (124.70)	2121.51 (107.57)	1807.14 (111.84)	2143.82 (142.83)	2074.05 (47.11)	0.128	0.163
Carbohydrate, g/day	307.21 (11.92)	299.38 (14.51)	318.84 (18.35)	304.50 (22.87)	298.32 (18.87)	381.77 (35.07)	310.47 (17.30)	307.43 (16.98)	272.22 (16.31)	321.42 (22.17)	312.50 (6.80)	0.323	0.898
Protein, g/day	66.34 (3.67)	78.55 (6.59)	72.33 (5.60)	78.05 (8.50)	71.77 (7.54)	87.01 (9.00)	86.44 (6.33)	82.23 (4.94)	65.96 (4.96)	79.42 (6.59)	77.49 (2.11)	0.046	0.201
Fat, g/day	43.89 (3.46)	59.54 (6.37)	47.38 (4.33)	45.93 (4.28)	53.83 (5.43)	68.73 (10.89)	61.30 (5.18)	58.15 (5.68)	48.89 (4.46)	56.95 (5.81)	54.93 (1.96)	0.038	0.061
Normal WC	n=902	n=705	n=631	n=598	n=623	n=420	n=456	n=494	n=469	n=440	n=5,738		
Age, yr	14.19 (0.10)	14.23 (0.13)	14.16 (0.14)	14.28 (0.12)	14.28 (0.11)	14.32 (0.14)	14.35 (0.14)	14.46 (0.12)	14.26 (0.14)	14.36 (0.15)	14.28 (0.04)	0.886	0.133
Sex (male) , %	53.79 (1.99)	55.81 (2.17)	54.85 (2.32)	55.96 (2.29)	51.38 (1.90)	53.37 (2.52)	53.33 (2.61)	54.14 (2.58)	52.75 (2.41)	50.37 (2.53)	53.69 (0.74)	0.814	0.156
Height SDS	0.24 (0.05)	0.22 (0.06)	0.11 (0.05)	0.23 (0.06)	0.22 (0.04)	0.10 (0.08)	0.09 (0.05)	0.17 (0.07)	0.27 (0.06)	0.23 (0.06)	0.19 (0.02)	0.211	0.905
Weight SDS	-0.10 (0.05)	-0.13 (0.05)	-0.25 (0.05)	-0.12 (0.05)	-0.05 (0.05)	-0.14 (0.08)	-0.10 (0.06)	-0.27 (0.07)	-0.10 (0.05)	-0.09 (0.06)	-0.14 (0.02)	0.151	0.916
BMI SDS	-0.27 (0.04)	-0.30 (0.05)	-0.38 (0.05)	-0.29 (0.04)	-0.20 (0.05)	-0.24 (0.07)	-0.18 (0.06)	-0.43 (0.06)	-0.29 (0.06)	-0.26 (0.06)	-0.29 (0.02)	0.094	0.810
BMI percentile												>0.999	0.412
Normal, %	88.21 (1.22)	88.06 (1.42)	89.03 (1.47)	88.34 (1.56)	87.30 (1.49)	88.46 (1.78)	89.20 (1.60)	89.85 (1.51)	89.23 (1.78)	89.52 (1.65)	88.68 (0.49)		
Overweight, %	8.79 (0.98)	8.78 (1.21)	7.65 (1.23)	8.86 (1.34)	9.91 (1.25)	8.75 (1.48)	7.89 (1.33)	7.35 (1.33)	8.36 (1.48)	7.18 (1.21)	8.38 (0.40)		
Obesity, %	3.00 (0.71)	3.17 (0.78)	3.32 (0.88)	2.80 (0.76)	2.79 (0.69)	2.79 (0.88)	2.92 (0.77)	2.79 (0.80)	2.41 (0.94)	3.31 (1.23)	2.94 (0.27)		
Glucose, mg/dL	88.85 (0.27)	88.62 (0.31)	88.36 (0.38)	88.92 (0.41)	90.42 (0.37)	91.16 (0.41)	91.09 (0.32)	91.29 (0.31)	90.99 (0.38)	91.31 (0.39)	89.97 (0.12)	<0.01	<0.01
Prediabetes, %	4.67 (0.89)	4.04 (0.87)	6.33 (1.39)	2.89 (0.70)	8.02 (1.27)	10.61 (1.58)	7.73 (1.33)	9.82 (1.51)	10.26 (1.77)	9.86 (1.40)	7.18 (0.41)	<0.01	<0.01
AST, IU/L	18.51 (0.20)	18.72 (0.29)	18.34 (0.24)	19.29 (0.45)	18.06 (0.23)	18.53 (0.34)	18.95 (0.27)	18.57 (0.34)	19.40 (0.56)	19.47 (0.35)	18.76 (0.11)	0.024	0.028
ALT, IU/L	13.63 (0.31)	13.87 (0.52)	13.07 (0.41)	14.28 (0.75)	13.78 (0.42)	13.56 (0.59)	13.60 (0.42)	13.43 (0.66)	14.74 (1.42)	14.23 (0.73)	13.80 (0.21)	0.885	0.446

NAFLD, %	6.66 (1.03)	5.38 (1.15)	4.92 (0.96)	6.97 (1.44)	5.95 (0.95)	5.97 (1.37)	6.81 (1.26)	8.52 (1.70)	6.09 (1.24)	6.68 (1.25)	6.36 (0.39)	0.750	0.305
Prediabetes and NAFLD, %	0.30 (0.17)	0.40 (0.21)	0.43 (0.33)	0.08 (0.08)	1.01 (0.43)	1.27 (0.65)	0.77 (0.42)	1.81 (0.76)	1.76 (0.75)	1.25 (0.56)	0.86 (0.15)	0.022	<0.01
Total cholesterol , mg/dL	157.19 (1.38)	155.92 (1.19)	156.31 (1.49)	158.07 (1.51)	156.74 (1.17)	156.68 (1.38)	158.89 (1.34)	163.01 (1.42)	165.43 (1.52)	163.94 (1.29)	158.99 (0.44)	<0.01	<0.01
HDL-C, mg/dL	49.65 (0.41)	49.81 (0.44)	52.35 (0.56)	52.02 (0.67)	52.56 (0.43)	52.51 (0.55)	52.10 (0.51)	52.72 (0.60)	52.68 (0.52)	52.03 (0.58)	51.76 (0.17)	<0.01	<0.01
LDL-C, mg/dL	90.08 (1.11)	90.15 (1.03)	90.08 (1.26)	90.11 (1.13)	88.68 (0.97)	88.24 (1.27)	92.07 (1.28)	93.98 (1.12)	96.82 (1.23)	95.47 (1.22)	91.40 (0.37)	<0.01	<0.01
Triglycerides, mg/dL	87.19 (2.33)	82.80 (2.87)	76.81 (2.19)	82.61 (2.97)	80.46 (2.16)	81.81 (3.02)	83.64 (3.09)	83.75 (2.37)	82.10 (3.02)	85.91 (2.56)	82.66 (0.84)	0.125	0.586
Energy, kcal/day	1976.98 (32.06)	2210.47 (47.53)	2200.17 (39.85)	2187.85 (46.56)	2185.97 (47.11)	2202.92 (56.26)	2232.58 (52.92)	2143.64 (41.90)	2136.12 (50.74)	2134.77 (55.11)	2158.18 (14.71)	<0.01	0.375
Carbohydrate, g/day	313.09 (5.36)	338.73 (6.99)	337.67 (6.04)	338.90 (6.47)	328.08 (6.32)	323.71 (8.26)	330.90 (7.19)	320.52 (6.40)	316.32 (7.35)	313.12 (8.25)	326.49 (2.16)	<0.01	0.026
Protein, g/day	68.88 (1.14)	77.57 (2.25)	80.80 (2.39)	80.55 (2.55)	78.49 (2.50)	79.89 (2.66)	79.42 (2.56)	79.70 (1.99)	77.95 (2.37)	77.28 (2.38)	77.90 (0.72)	<0.01	0.075
Fat, g/day	49.93 (1.34)	59.79 (2.08)	57.92 (1.68)	57.57 (2.04)	58.78 (1.94)	61.34 (2.18)	62.18 (2.30)	57.64 (1.69)	59.18 (2.07)	60.31 (2.12)	58.20 (0.61)	<0.01	<0.01

Continuous variables are presented as the mean (standard error) and categorical data as the percentage (standard error) *with Italic.* For calculating *p* value, analysis of variance was used to compare the mean values of the continuous variables and the Rao–Scott Chi-squared test was used to compare categorical variables. Linear trend analysis was performed using coefficients of orthogonal polynomials to calculate p for trend. SDS: standard deviation score; BMI: body mass index; AST: aspartate aminotransferase; ALT: alanine aminotransferase; NAFLD: non-alcoholic fatty liver disease; HDL-C: high-density lipoprotein cholesterol; LDL-C: low-density lipoprotein cholesterol; TyG index: triglyceride-glucose index; WC: waist circumference.

Table S2. Trend of characteristics of participants according to sex

Variable	2009	2010	2011	2012	2013	2014	2015	2016	2017	2018	*Total*	*P*	*P* for trend
**Male**	n=501	n=419	n=365	n=340	n=347	n=252	n=293	n=302	n=259	n=269	n=3347		
Age, yr	14.26 (0.13)	14.20 (0.15)	14.28 (0.17)	14.26 (0.13)	14.29 (0.14)	14.30 (0.17)	14.42 (0.18)	14.60 (0.16)	14.38 (0.19)	14.40 (0.16)	14.33 (0.05)	0.840	0.103
Height SDS	0.37 (0.06)	0.29 (0.07)	0.19 (0.07)	0.24 (0.08)	0.28 (0.05)	0.19 (0.08)	0.22 (0.08)	0.20 (0.08)	0.34 (0.09)	0.32 (0.06)	0.26 (0.02)	0.485	0.803
Weight SDS	0.06 (0.06)	0.05 (0.08)	-0.05 (0.08)	0.06 (0.08)	0.16 (0.08)	0.08 (0.10)	0.21 (0.08)	-0.01 (0.10)	0.11 (0.08)	0.23 (0.08)	0.08 (0.03)	0.283	0.082
BMI SDS	-0.15 (0.05)	-0.12 (0.08)	-0.18 (0.08)	-0.08 (0.08)	0.01 (0.09)	-0.04 (0.10)	0.14 (0.10)	-0.13 (0.10)	-0.08 (0.09)	0.08 (0.09)	-0.06 (0.03)	0.137	0.030
BMI percentile												0.252	0.056
Normal, %	83.52 (1.67)	79.64 (2.31)	80.84 (2.43)	79.71 (2.83)	77.94 (2.56)	81.01 (2.84)	76.59 (2.82)	75.97 (2.69)	83.66 (2.34)	78.95 (2.56)	79.87 (0.79)		
Overweight, %	10.23 (1.40)	11.64 (1.77)	6.94 (1.49)	9.66 (2.09)	11.42 (1.88)	8.20 (1.88)	9.96 (1.85)	10.98 (1.92)	6.28 (1.44)	8.25 (1.92)	9.39 (0.56)		
Obesity, %	6.25 (0.98)	8.72 (1.59)	12.22 (2.10)	10.63 (2.32)	10.63 (1.82)	10.80 (2.35)	13.45 (2.16)	13.05 (2.24)	10.05 (2.00)	12.80 (2.12)	10.74 (0.63)		
WC, cm	71.18 (0.40)	71.07 (0.63)	71.08 (0.65)	71.32 (0.70)	71.55 (0.76)	72.16 (0.80)	74.60 (0.65)	72.85 (0.73)	72.04 (0.69)	73.34 (0.72)	72.02 (0.21)	<0.01	<0.01
Abdominal obesity, %	4.85 (0.97)	5.93 (1.24)	9.56 (1.85)	7.63 (1.78)	8.73 (1.70)	8.76 (2.24)	13.50 (1.99)	11.85 (2.06)	9.58 (1.98)	12.33 (2.15)	9.07 (0.57)	0.011	<0.01
Glucose, mg/dL	89.65 (0.35)	89.29 (0.38)	88.96 (0.42)	88.88 (0.48)	91.17 (0.47)	93.01 (0.53)	92.06 (0.40)	92.08 (0.40)	92.98 (0.46)	92.29 (0.50)	90.87 (0.15)	<0.01	<0.01
Prediabetes,%	6.32 (1.35)	5.03 (1.30)	6.49 (1.65)	3.55 (1.03)	9.45 (1.93)	15.68 (2.45)	10.38 (1.98)	12.19 (2.13)	16.12 (2.78)	12.62 (2.13)	9.38 (0.60)	<0.01	<0.01
AST, IU/L	19.67 (0.31)	20.20 (0.41)	20.64 (0.46)	21.35 (0.77)	20.10 (0.44)	20.37 (0.53)	21.87 (1.18)	20.17 (0.40)	21.60 (0.96)	21.83 (0.59)	20.74 (0.20)	0.046	0.010
ALT, IU/L	16.60 (0.69)	17.28 (0.85)	18.82 (1.40)	17.57 (1.28)	18.11 (0.92)	17.55 (1.06)	19.69 (2.22)	17.27 (0.97)	19.88 (2.52)	20.80 (1.56)	18.27 (0.44)	0.408	0.050
NAFLD, %	9.62 (1.53)	10.01 (1.89)	12.84 (1.90)	12.37 (2.62)	13.82 (2.09)	13.61 (2.82)	13.70 (2.25)	15.49 (2.80)	12.06 (2.21)	18.01 (2.61)	12.97 (0.72)	0.326	<0.01
Total cholesterol, mg/dL	153.03 (1.90)	150.98 (1.59)	154.06 (1.98)	155.17 (1.75)	151.77 (1.53)	151.77 (1.76)	156.91 (1.76)	158.92 (1.72)	162.96 (1.75)	162.97 (1.89)	1.79 (0.27)	<0.01	<0.01
HDL-C, mg/dL	47.67 (0.47)	48.27 (0.55)	50.24 (0.71)	49.74 (0.70)	50.13 (0.58)	50.63 (0.66)	50.99 (0.73)	50.60 (0.71)	50.22 (0.62)	49.58 (0.68)	1.05 (0.19)	<0.01	<0.01
LDL-C, mg/dL	87.65 (1.58)	86.54 (1.33)	90.00 (1.81)	89.24 (1.37)	85.87 (1.24)	84.09 (1.51)	91.44 (1.62)	92.20 (1.41)	96.35 (1.46)	96.57 (1.76)	4.97 (0.45)	<0.01	<0.01
Triglycerides, mg/dL	88.27 (3.15)	84.65 (3.65)	78.23 (3.06)	84.22 (3.68)	82.95 (3.25)	89.95 (5.05)	86.45 (4.15)	82.15 (3.08)	85.67 (4.60)	88.64 (3.53)	0.50 (0.13)	0.415	0.442
Prediabetes and NAFLD, %	0.83 (0.41)	1.22 (0.52)	0.71 (0.55)	0.12 (0.12)	2.02 (0.84)	3.02 (1.27)	1.33 (0.70)	2.54 (1.16)	4.17 (1.46)	2.98 (1.14)	1.79 (0.27)	<0.01	<0.01
Prediabetes and abdominal obesity, %	0.80 (0.43)	0.66 (0.40)	0.00 (0.00)	0.00 (0.00)	1.02 (0.60)	1.14 (0.69)	1.64 (0.79)	1.71 (0.80)	2.53 (1.06)	1.65 (0.81)	1.05 (0.19)	0.012	<0.01
NAFLD and abdominal obesity, %	2.44 (0.66)	2.96 (0.83)	6.49 (1.62)	3.78 (1.32)	5.64 (1.49)	4.39 (1.99)	6.59 (1.37)	4.94 (1.40)	5.24 (1.53)	8.37 (1.86)	4.97 (0.45)	0.105	<0.01
Prediabetes, NAFLD, and abdominal obesity, %	0.47 (0.33)	0.66 (0.40)	0.00 (0.00)	0.00 (0.00)	0.42 (0.42)	0.84 (0.62)	0.08 (0.08)	0.43 (0.43)	1.16 (0.73)	1.25 (0.73)	0.50 (0.13)	0.133	0.147
Energy, kcal/day	2161.80 (38.44)	2442.30 (66.14)	2435.99 (59.95)	2376.68 (62.63)	2435.62 (66.82)	2526.81 (84.02)	2484.13 (68.30)	2408.52 (58.48)	2363.85 (66.03)	2393.90 (75.85)	2398.53 (20.48)	<0.01	0.102
Carbohydrate, g/day	341.14 (6.01)	369.73 (8.93)	372.07 (8.90)	369.36 (8.70)	365.41 (8.80)	368.58 (12.40)	359.94 (9.75)	357.51 (8.60)	346.22 (9.59)	350.02 (10.69)	360.32 (2.92)	0.044	0.244
Protein, g/day	75.62 (1.46)	87.66 (3.05)	91.32 (3.75)	88.42 (3.20)	88.09 (3.25)	92.79 (4.03)	90.64 (3.02)	90.41 (2.73)	87.07 (3.13)	88.09 (3.30)	87.82 (1.00)	<0.01	0.029
Fat, g/day	54.50 (1.71)	66.37 (3.01)	64.32 (2.44)	61.13 (2.76)	65.11 (2.98)	70.68 (3.09)	70.76 (2.91)	64.67 (2.68)	66.01 (2.88)	67.25 (3.01)	64.73 (0.88)	<0.01	<0.01
**Female**	n=464	n=352	n=334	n=303	n=325	n=214	n=242	n=267	n=258	n=221	n=2980		
Age, yr	14.11 (0.13)	14.22 (0.21)	14.11 (0.16)	14.35 (0.20)	14.32 (0.15)	14.48 (0.21)	14.30 (0.17)	14.37 (0.16)	14.37 (0.18)	14.42 (0.21)	14.30 (0.06)	0.844	0.112
Height SDS	0.19 (0.06)	0.20 (0.07)	0.13 (0.07)	0.33 (0.09)	0.22 (0.07)	0.14 (0.11)	0.12 (0.08)	0.30 (0.07)	0.21 (0.07)	0.28 (0.08)	0.21 (0.02)	0.613	0.541
Weight SDS	-0.00 (0.07)	0.04 (0.08)	0.10 (0.08)	0.08 (0.09)	0.11 (0.06)	0.15 (0.12)	0.29 (0.10)	0.11 (0.09)	0.16 (0.09)	0.10 (0.08)	0.11 (0.03)	0.644	0.091
BMI SDS	-0.12 (0.08)	-0.07 (0.08)	0.02 (0.09)	-0.09 (0.08)	-0.00 (0.06)	0.10 (0.10)	0.28 (0.10)	-0.04 (0.09)	0.06 (0.09)	-0.05 (0.09)	0.00 (0.03)	0.102	0.099
BMI percentile												0.470	0.070
Normal, %	83.54 (2.12)	83.45 (2.75)	78.84 (2.62)	82.95 (2.93)	84.81 (2.11)	79.56 (3.31)	76.91 (2.69)	81.99 (2.60)	77.14 (2.88)	82.12 (2.94)	81.22 (0.85)		
Overweight, %	9.57 (1.50)	8.19 (1.86)	10.09 (1.86)	9.19 (2.07)	8.51 (1.51)	9.77 (2.13)	7.97 (1.74)	7.20 (1.85)	10.57 (2.16)	7.46 (1.72)	8.88 (0.58)		
Obesity, %	6.89 (1.78)	8.36 (2.13)	11.07 (2.12)	7.86 (2.06)	6.69 (1.49)	10.66 (2.38)	15.12 (2.53)	10.81 (2.04)	12.29 (2.32)	10.42 (2.41)	9.90 (0.67)		

WC, cm	66.55 (0.51)	67.13 (0.69)	67.43 (0.60)	66.82 (0.59)	66.85 (0.48)	68.26 (0.71)	70.06 (0.67)	68.22 (0.63)	67.74 (0.66)	66.98 (0.60)	67.56 (0.20)	0.004	0.020
Abdominal obesity, %	7.24 (1.36)	10.77 (2.33)	12.61 (1.99)	8.88 (2.17)	7.48 (1.69)	11.84 (2.27)	15.54 (2.50)	14.48 (2.29)	11.00 (2.39)	8.59 (2.30)	10.76 (0.68)	0.073	0.187
Glucose, mg/dL	87.98 (0.36)	88.17 (0.39)	88.19 (0.49)	88.83 (0.48)	89.65 (0.44)	89.62 (0.49)	90.65 (0.61)	90.72 (0.46)	89.04 (0.48)	90.79 (0.48)	89.29 (0.15)	<0.01	<0.01
Prediabetes,%	3.79 (1.16)	4.38 (1.12)	6.64 (1.71)	2.42 (0.85)	6.76 (1.57)	5.03 (1.65)	7.84 (1.96)	8.88 (2.06)	4.70 (1.32)	8.18 (1.78)	5.80 (0.50)	0.046	0.014
AST, IU/L	17.55 (0.24)	17.16 (0.27)	17.05 (0.29)	17.26 (0.48)	16.60 (0.25)	16.86 (0.32)	17.90 (0.40)	17.28 (0.47)	17.96 (0.37)	18.75 (0.63)	17.42 (0.12)	<0.01	<0.01
ALT, IU/L	12.01 (0.35)	11.20 (0.30)	11.75 (0.49)	12.31 (0.88)	11.79 (0.34)	11.42 (0.50)	12.87 (0.67)	12.53 (0.98)	13.17 (0.77)	13.62 (1.57)	12.23 (0.24)	0.159	0.047
NAFLD, %	6.52 (1.55)	2.56 (0.89)	4.63 (1.33)	5.09 (1.79)	4.02 (1.10)	3.21 (1.55)	8.21 (2.14)	7.95 (2.02)	9.35 (1.97)	5.75 (1.77)	5.67 (0.52)	0.053	0.032
Total cholesterol, mg/dL	163.06 (1.43)	163.57 (1.66)	162.31 (1.85)	164.35 (2.22)	163.58 (1.57)	165.02 (1.99)	164.39 (1.90)	169.38 (1.82)	171.22 (2.01)	167.87 (1.75)	0.53 (0.16)	0.006	<0.01
HDL-C, mg/dL	51.18 (0.57)	50.75 (0.60)	52.79 (0.72)	53.93 (0.78)	54.34 (0.58)	53.45 (0.76)	51.74 (0.62)	53.58 (0.80)	53.60 (0.68)	52.99 (0.82)	1.45 (0.26)	<0.01	0.017
LDL-C, mg/dL	94.15 (1.28)	96.65 (1.50)	94.47 (1.67)	93.61 (1.95)	93.24 (1.34)	95.81 (1.77)	97.26 (1.78)	98.32 (1.55)	100.72 (1.71)	97.52 (1.57)	2.52 (0.34)	0.018	<0.01
Triglycerides, mg/dL	88.68 (2.74)	85.56 (3.71)	84.76 (3.39)	89.34 (4.01)	82.15 (2.46)	80.62 (3.15)	88.16 (3.38)	90.94 (3.55)	86.51 (3.37)	91.85 (3.53)	0.34 (0.14)	0.207	0.330
Prediabetes and NAFLD, %	0.19 (0.19)	0.13 (0.13)	0.33 (0.33)	0.30 (0.30)	0.50 (0.35)	0.08 (0.08)	1.06 (1.06)	1.37 (0.78)	0.00 (0.00)	1.58 (0.87)	0.53 (0.16)	0.041	0.018
Prediabetes and abdominal obesity, %	0.69 (0.47)	1.45 (0.88)	2.00 (0.93)	0.90 (0.52)	0.53 (0.37)	1.05 (0.71)	3.60 (1.50)	2.56 (1.03)	0.31 (0.31)	1.63 (0.95)	1.45 (0.26)	0.094	0.400
NAFLD and abdominal obesity, %	1.30 (0.56)	0.14 (0.14)	2.55 (0.94)	1.44 (0.85)	1.45 (0.73)	2.12 (1.03)	3.82 (1.63)	4.14 (1.49)	5.37 (1.53)	3.65 (1.42)	2.52 (0.34)	<0.01	<0.01
Prediabetes, NAFLD, and abdominal obesity, %	0.00 (0.00)	0.00 (0.00)	0.33 (0.33)	0.30 (0.30)	0.28 (0.28)	0.08 (0.08)	1.06 (1.06)	0.41 (0.41)	0.00 (0.00)	1.10 (0.79)	0.34 (0.14)	0.059	0.059
Energy, kcal/day	1755.57 (43.27)	1903.74 (52.83)	1871.37 (42.46)	1910.80 (56.88)	1889.39 (59.31)	1913.86 (61.26)	1936.02 (54.55)	1834.03 (44.24)	1815.03 (52.52)	1862.54 (63.29)	1867.43 (16.77)	0.248	0.795
Carbohydrate, g/day	280.50 (7.23)	294.68 (7.60)	292.80 (6.39)	295.02 (7.78)	283.08 (8.05)	286.60 (9.09)	292.25 (7.99)	274.47 (7.03)	273.97 (8.00)	275.88 (9.83)	285.12 (2.49)	0.274	0.054
Protein, g/day	60.90 (1.58)	65.70 (2.22)	66.43 (1.81)	70.33 (3.30)	67.07 (3.91)	67.15 (2.81)	69.05 (2.92)	68.14 (2.37)	65.34 (2.41)	66.31 (2.94)	66.54 (0.85)	0.116	0.260
Fat, g/day	43.96 (1.72)	51.89 (2.58)	47.89 (1.82)	51.12 (2.37)	51.17 (2.39)	52.61 (2.82)	52.34 (2.20)	49.74 (1.96)	49.46 (2.00)	52.24 (2.79)	50.08 (0.72)	0.051	0.082

Continuous variables are presented as the mean (standard error) and categorical data as the percentage (standard error) *with Italic.* For calculating *p* value, analysis of variance was used to compare the mean values of the continuous variables and the Rao–Scott Chi-squared test was used to compare categorical variables. Linear trend analysis was performed using coefficients of orthogonal polynomials to calculate p for trend. SDS: standard deviation score; BMI: body mass index; WC: waist circumference; AST: aspartate aminotransferase; ALT: alanine aminotransferase; NAFLD: non-alcoholic fatty liver disease; HDL: high-density lipoprotein cholesterol; LDL-C: low-density lipoprotein cholesterol; TyG index: triglyceride-glucose index.

Table S3. Trend of prediabetes of subjects according to sex and age classification

Variable	2009	2010	2011	2012	2013	2014	2015	2016	2017	2018	*Total*	*P*	*P* for trend
**Male**													
10-12 y	n=168	n=174	n=140	n=119	n=132	n=78	n=100	n=97	n=94	n=102	n=1204		
BMI SDS	0.02 (0.09)	0.05 (0.12)	-0.14 (0.11)	-0.06 (0.11)	0.09 (0.11)	-0.17 (0.15)	0.20 (0.16)	-0.03 (0.16)	-0.16 (0.13)	0.06 (0.12)	-0.02 (0.04)	0.629	0.972
BMI percentile												0.484	0.639
Normal, %	81.88 (3.14)	75.95 (3.96)	81.32 (4.25)	85.52 (3.28)	79.02 (3.89)	85.38 (4.01)	75.68 (4.84)	74.13 (5.21)	86.57 (3.62)	76.76 (3.83)	80.29 (1.28)		
Overweight, %	11.18 (2.79)	13.38 (3.14)	11.05 (3.22)	5.01 (1.82)	14.21 (3.29)	9.05 (3.01)	10.46 (3.18)	12.59 (3.91)	8.58 (3.04)	12.35 (3.17)	10.81 (0.99)		
Obesity, %	6.94 (1.97)	10.67 (2.83)	7.63 (2.36)	9.47 (2.83)	6.77 (2.24)	5.57 (2.91)	13.86 (4.05)	13.28 (3.59)	4.85 (2.46)	10.89 (3.31)	8.90 (0.90)		
WC, cm	67.40 (0.74)	67.22 (0.83)	65.89 (0.97)	66.54 (0.98)	67.17 (0.93)	65.84 (1.13)	70.30 (1.40)	67.79 (1.24)	65.81 (1.06)	68.17 (1.05)	67.14 (0.32)	0.246	0.331
Abdominal obesity, %	7.98 (2.12)	8.24 (2.62)	7.13 (2.39)	6.44 (2.38)	7.17 (2.24)	3.44 (2.01)	17.09 (4.33)	8.57 (2.74)	4.85 (2.46)	11.63 (3.28)	8.11 (0.85)	0.080	0.421
Glucose, mg/dL	91.56 (0.45)	90.70 (0.63)	90.37 (0.55)	90.99 (0.56)	93.33 (0.63)	94.75 (0.75)	93.50 (0.64)	93.69 (0.58)	94.70 (0.87)	94.48 (0.66)	92.57 (0.21)	<0.01	<0.01
Prediabetes, %	8.10 (2.74)	7.29 (2.44)	9.56 (3.34)	6.73 (2.72)	12.04 (3.50)	18.67 (4.06)	17.46 (4.07)	13.30 (3.50)	18.43 (4.91)	21.17 (4.39)	12.61 (1.13)	0.019	<0.01
AST, IU/L	23.00 (0.78)	22.68 (0.60)	23.82 (0.90)	24.68 (1.90)	22.64 (0.78)	23.21 (0.77)	28.73 (4.01)	22.31 (0.43)	22.69 (0.74)	24.58 (0.81)	23.75 (0.46)	0.303	0.299
ALT, IU/L	19.14 (1.98)	18.58 (1.39)	20.73 (2.51)	18.26 (2.71)	17.91 (1.66)	17.05 (1.73)	26.97 (7.65)	15.26 (0.82)	16.35 (1.67)	20.04 (1.63)	19.02 (0.89)	0.100	0.858
NAFLD, %	11.57 (2.79)	9.31 (2.50)	10.54 (2.91)	11.62 (6.07)	10.16 (2.86)	8.72 (3.76)	19.94 (4.37)	8.70 (2.84)	7.35 (2.95)	18.81 (3.81)	11.47 (1.16)	0.286	0.298
13-15 y	n=200	n=146	n=136	n=134	n=123	n=103	n=105	n=115	n=86	n=93	n=1241		
BMI SDS	-0.14 (0.07)	-0.11 (0.13)	-0.03 (0.14)	-0.03 (0.14)	-0.16 (0.17)	0.00 (0.13)	0.30 (0.13)	-0.02 (0.16)	0.10 (0.16)	0.05 (0.16)	-0.02 (0.04)	0.288	0.063
BMI percentile												0.095	0.196
Normal, %	81.98 (2.62)	78.63 (3.81)	75.63 (4.35)	72.94 (5.03)	83.18 (3.83)	82.59 (3.99)	73.70 (5.04)	72.25 (4.87)	79.18 (4.71)	81.24 (4.54)	78.09 (1.37)		
Overweight, %	10.24 (2.13)	14.36 (3.35)	7.75 (2.77)	15.19 (3.94)	8.40 (2.55)	8.12 (3.16)	11.99 (3.56)	10.93 (3.61)	7.25 (2.48)	1.20 (0.97)	9.85 (0.98)		
Obesity, %	7.77 (1.85)	7.02 (2.27)	16.61 (3.91)	11.88 (4.20)	8.42 (2.92)	9.30 (3.02)	14.31 (4.12)	16.82 (4.04)	13.57 (4.02)	17.56 (4.44)	12.06 (1.12)		
WC, cm	71.61 (0.59)	71.57 (1.10)	72.51 (1.14)	72.33 (1.12)	71.18 (1.23)	72.91 (1.03)	75.21 (0.96)	73.55 (1.17)	73.49 (1.27)	73.33 (1.18)	72.66 (0.34)	0.114	0.015
Abdominal obesity, %	4.01 (1.36)	5.25 (2.07)	12.21 (3.55)	9.04 (3.40)	6.97 (2.82)	8.42 (2.94)	10.54 (3.35)	11.30 (3.46)	12.71 (3.91)	12.87 (3.67)	9.05 (0.98)	0.330	0.012
Glucose, mg/dL	90.19 (0.50)	89.41 (0.59)	89.54 (0.54)	89.43 (0.90)	91.55 (0.67)	93.17 (0.76)	92.69 (0.64)	92.42 (0.78)	93.67 (0.69)	92.10 (0.82)	91.21 (0.23)	<0.01	<0.01
Prediabetes, %	8.13 (2.30)	5.04 (2.33)	6.23 (2.44)	3.78 (1.45)	11.12 (3.33)	16.26 (3.90)	10.33 (3.08)	14.98 (4.21)	19.19 (4.98)	12.03 (3.62)	10.14 (1.01)	<0.01	<0.01
AST, IU/L	18.83 (0.35)	19.11 (0.46)	18.87 (0.57)	19.93 (0.87)	18.43 (0.58)	19.31 (0.77)	19.51 (0.50)	19.93 (0.63)	24.45 (2.81)	20.75 (0.68)	19.76 (0.30)	0.114	0.006
ALT, IU/L	15.01 (0.66)	14.99 (1.06)	15.56 (1.20)	16.55 (2.44)	14.46 (1.03)	15.52 (1.35)	15.64 (0.76)	17.75 (2.07)	25.72 (7.61)	17.16 (1.62)	16.59 (0.77)	0.741	0.073
NAFLD, %	7.73 (1.97)	8.31 (2.63)	12.12 (3.69)	11.24 (3.46)	8.68 (2.95)	8.15 (3.12)	9.70 (3.00)	12.86 (3.87)	15.06 (4.34)	11.54 (3.71)	10.39 (1.04)	0.850	0.179
16-18 y	n=133	n=99	n=89	n=87	n=92	n=71	n=88	n=90	n=79	n=74	n=902		
BMI SDS	-0.29 (0.11)	-0.27 (0.14)	-0.36 (0.17)	-0.16 (0.14)	0.13 (0.17)	0.02 (0.22)	-0.05 (0.15)	-0.28 (0.17)	-0.17 (0.16)	0.13 (0.17)	-0.14 (0.05)	0.278	0.068
BMI percentile												0.472	0.158
Normal, %	86.59 (3.46)	83.79 (3.91)	85.81 (3.88)	82.39 (4.81)	71.80 (5.28)	76.10 (5.81)	79.74 (4.05)	79.76 (4.39)	85.08 (4.07)	78.66 (5.04)	81.22 (1.42)		
Overweight, %	9.46 (2.94)	7.61 (2.93)	2.57 (1.63)	7.39 (3.54)	12.18 (3.95)	7.64 (3.36)	7.83 (2.77)	10.12 (3.20)	3.96 (2.12)	10.97 (3.80)	7.90 (0.98)		
Obesity, %	3.95 (1.75)	8.60 (2.78)	11.62 (3.61)	10.22 (3.58)	16.02 (4.07)	16.27 (5.12)	12.43 (3.27)	10.12 (3.44)	10.95 (3.62)	10.37 (3.51)	10.88 (1.12)		
WC, cm	73.74 (0.74)	73.97 (0.93)	74.05 (1.15)	73.98 (1.05)	75.51 (1.25)	76.07 (1.42)	76.94 (0.99)	75.19 (1.24)	75.21 (1.09)	76.97 (1.28)	75.09 (0.35)	0.177	<0.01
Abdominal obesity, %	3.29 (1.75)	4.55 (1.78)	8.89 (3.36)	7.06 (3.40)	11.78 (3.54)	13.09 (4.98)	13.68 (3.34)	14.10 (3.84)	10.46 (3.50)	12.39 (4.17)	9.80 (1.09)	0.223	<0.01
Glucose, mg/dL	87.49 (0.61)	87.94 (0.70)	87.16 (0.80)	86.65 (0.63)	89.03 (0.76)	91.56 (0.97)	90.56 (0.64)	90.92 (0.71)	91.27 (0.79)	90.89 (0.78)	89.27 (0.25)	<0.01	<0.01
Prediabetes, %	2.84 (1.68)	3.04 (2.08)	4.12 (2.53)	0.80 (0.56)	5.65 (2.84)	12.85 (4.06)	5.67 (2.77)	9.49 (3.43)	12.18 (4.48)	7.08 (2.97)	6.24 (0.94)	0.029	<0.01
AST, IU/L	17.93(0.39)	19.04(0.84)	19.75(0.94)	20.26(1.48)	19.70(0.85)	19.39(1.03)	19.34(0.74)	19.15(0.67)	18.68(0.67)	20.77(1.13)	19.38 (0.29)	0.197	0.324
ALT, IU/L	16.35(0.86)	18.24(1.68)	20.54(3.36)	18.12(1.72)	21.93(1.99)	20.05(2.05)	18.36(1.78)	18.05(1.34)	17.85(1.80)	24.21(3.14)	19.27 (0.66)	0.156	0.203
NAFLD, %	10.19 (2.77)	12.20 (3.63)	15.55 (4.15)	14.17 (4.58)	21.98 (4.75)	23.02 (5.87)	13.03 (3.65)	21.26 (4.98)	13.02 (3.87)	22.59 (5.10)	16.52 (1.40)	0.251	0.049
**Female**													
10-12 y	n=172	n=119	n=130	n=127	n=107	n=64	n=83	n=86	n=96	n=87	n=1071		
BMI SDS	0.02 (0.09)	0.05 (0.12)	-0.14 (0.11)	-0.06 (0.11)	0.09 (0.11)	-0.17 (0.15)	0.20 (0.16)	-0.03 (0.16)	-0.16 (0.13)	0.06 (0.12)	-0.14 (0.04)	0.629	0.972
BMI percentile												0.484	0.639
Normal, %	81.88 (3.14)	75.95 (3.96)	81.32 (4.25)	85.52 (3.28)	79.02 (3.89)	85.38 (4.01)	75.68 (4.84)	74.13 (5.21)	86.57 (3.62)	76.76 (3.83)	85.07 (1.24)		

Overweight, %	11.18 (2.79)	13.38 (3.14)	11.05 (3.22)	5.01 (1.82)	14.21 (3.29)	9.05 (3.01)	10.46 (3.18)	12.59 (3.91)	8.58 (3.04)	12.35 (3.17)	8.45 (0.92)		
Obesity, %	6.94 (1.97)	10.67 (2.83)	7.63 (2.36)	9.47 (2.83)	6.77 (2.24)	5.57 (2.91)	13.86 (4.05)	13.28 (3.59)	4.85 (2.46)	10.89 (3.31)	6.48 (0.93)		
WC, cm	67.40 (0.74)	67.22 (0.83)	65.89 (0.97)	66.54 (0.98)	67.17 (0.93)	65.84 (1.13)	70.30 (1.40)	67.79 (1.24)	65.81 (1.06)	68.17 (1.05)	63.66 (0.29)	0.246	0.331
Abdominal obesity, %	7.98 (2.12)	8.24 (2.62)	7.13 (2.39)	6.44 (2.38)	7.17 (2.24)	3.44 (2.01)	17.09 (4.33)	8.57 (2.74)	4.85 (2.46)	11.63 (3.28)	8.98 (1.06)	0.080	0.421
Glucose, mg/dL	89.50 (0.46)	90.30 (0.71)	89.14 (0.64)	90.05 (0.67)	91.93 (0.70)	91.59 (0.83)	92.51 (0.68)	92.78 (0.68)	92.03 (0.70)	93.34 (0.77)	91.18 (0.22)	<0.01	<0.01
Prediabetes, %	5.71 (2.43)	7.41 (2.28)	5.36 (2.34)	3.24 (1.78)	10.70 (3.48)	6.02 (2.77)	8.98 (3.42)	12.51 (4.06)	11.43 (3.47)	13.96 (3.73)	8.32 (0.96)	0.181	<0.01
AST, IU/L	19.66 (0.36)	20.22 (0.53)	19.34 (0.46)	18.29 (0.32)	18.92 (0.47)	19.51 (0.52)	20.02 (0.62)	19.63 (0.57)	20.22 (0.49)	21.99 (0.96)	19.75 (0.17)	<0.01	0.010
ALT, IU/L	12.52 (0.40)	12.54 (0.53)	11.76 (0.40)	11.61 (0.40)	12.95 (0.70)	11.61 (0.47)	12.44 (0.63)	12.53 (0.76)	12.42 (0.58)	14.23 (1.27)	12.44 (0.20)	0.340	0.181
NAFLD, %	11.57 (2.79)	9.31 (2.50)	10.54 (2.91)	11.62 (6.07)	10.16 (2.86)	8.72 (3.76)	19.94 (4.37)	8.70 (2.84)	7.35 (2.95)	18.81 (3.81)	4.95 (0.75)	0.286	0.298
13-15 y	n=159	n=149	N=106	n=98	n=111	n=77	n=80	n=109	n=83	n=67	n=1033		
BMI SDS	-0.14 (0.07)	-0.11 (0.13)	-0.03 (0.14)	-0.03 (0.14)	-0.16 (0.17)	0.00 (0.13)	0.30 (0.13)	-0.02 (0.16)	0.10 (0.16)	0.05 (0.16)	-0.02 (0.04)	0.288	0.063
BMI percentile												0.095	0.196
Normal, %	81.98 (2.62)	78.63 (3.81)	75.63 (4.35)	72.94 (5.03)	83.18 (3.83)	82.59 (3.99)	73.70 (5.04)	72.25 (4.87)	79.18 (4.71)	81.24 (4.54)	82.43 (1.39)		
Overweight, %	10.24 (2.13)	14.36 (3.35)	7.75 (2.77)	15.19 (3.94)	8.40 (2.55)	8.12 (3.16)	11.99 (3.56)	10.93 (3.61)	7.25 (2.48)	1.20 (0.97)	8.83 (0.99)		
Obesity, %	7.77 (1.85)	7.02 (2.27)	16.61 (3.91)	11.88 (4.20)	8.42 (2.92)	9.30 (3.02)	14.31 (4.12)	16.82 (4.04)	13.57 (4.02)	17.56 (4.44)	8.73 (1.04)		
WC, cm	71.61 (0.59)	71.57 (1.10)	72.51 (1.14)	72.33 (1.12)	71.18 (1.23)	72.91 (1.03)	75.21 (0.96)	73.55 (1.17)	73.49 (1.27)	73.33 (1.18)	67.93 (0.28)	0.114	0.015
Abdominal obesity, %	4.01 (1.36)	5.25 (2.07)	12.21 (3.55)	9.04 (3.40)	6.97 (2.82)	8.42 (2.94)	10.54 (3.35)	11.30 (3.46)	12.71 (3.91)	12.87 (3.67)	8.81 (1.01)	0.330	0.012
Glucose, mg/dL	87.97 (0.48)	87.94 (0.62)	89.22 (0.83)	89.78 (0.57)	89.56 (0.76)	89.63 (0.80)	89.95 (0.63)	90.60 (0.66)	88.77 (0.77)	90.20 (0.97)	89.28 (0.23)	0.022	0.014
Prediabetes, %	1.52 (0.93)	4.03 (1.62)	11.18 (4.11)	4.14 (2.10)	5.96 (2.41)	5.61 (2.31)	5.95 (2.38)	5.09 (2.16)	1.02 (1.02)	7.82 (3.21)	5.19 (0.77)	0.070	0.725
AST, IU/L	16.78(0.30)	16.37(0.43)	16.04(0.25)	15.91(0.59)	16.11(0.28)	16.15(0.42)	17.21(0.64)	16.46(0.40)	17.01(0.80)	17.38(0.70)	16.52 (0.15)	0.366	0.101
ALT, IU/L	11.69(0.64)	10.57(0.43)	11.17(0.47)	10.85(0.87)	11.26(0.44)	10.23(0.40)	11.97(1.03)	11.32(0.72)	12.63(1.92)	11.56(0.99)	11.29 (0.26)	0.496	0.379
NAFLD, %	7.73 (1.97)	8.31 (2.63)	12.12 (3.69)	11.24 (3.46)	8.68 (2.95)	8.15 (3.12)	9.70 (3.00)	12.86 (3.87)	15.06 (4.34)	11.54 (3.71)	4.54 (0.86)	0.850	0.179
16-18 y	n=133	n=84	n=98	n=78	n=107	n=73	n=79	n=78	n=79	n=67	n=876		
BMI SDS	-0.29 (0.11)	-0.27 (0.14)	-0.36 (0.17)	-0.16 (0.14)	0.13 (0.17)	0.02 (0.22)	-0.05 (0.15)	-0.28 (0.17)	-0.17 (0.16)	0.13 (0.17)	0.13 (0.05)	0.278	0.068
BMI percentile												0.472	0.158
Normal, %	86.59 (3.46)	83.79 (3.91)	85.81 (3.88)	82.39 (4.81)	71.80 (5.28)	76.10 (5.81)	79.74 (4.05)	79.76 (4.39)	85.08 (4.07)	78.66 (5.04)	77.30 (1.60)		
Overweight, %	9.46 (2.94)	7.61 (2.93)	2.57 (1.63)	7.39 (3.54)	12.18 (3.95)	7.64 (3.36)	7.83 (2.77)	10.12 (3.20)	3.96 (2.12)	10.97 (3.80)	9.25 (1.06)		
Obesity, %	3.95 (1.75)	8.60 (2.78)	11.62 (3.61)	10.22 (3.58)	16.02 (4.07)	16.27 (5.12)	12.43 (3.27)	10.12 (3.44)	10.95 (3.62)	10.37 (3.51)	13.46 (1.31)		
WC, cm	73.74 (0.74)	73.97 (0.93)	74.05 (1.15)	73.98 (1.05)	75.51 (1.25)	76.07 (1.42)	76.94 (0.99)	75.19 (1.24)	75.21 (1.09)	76.97 (1.28)	70.15 (0.34)	0.177	0.006
Abdominal obesity, %	3.29 (1.75)	4.55 (1.78)	8.89 (3.36)	7.06 (3.40)	11.78 (3.54)	13.09 (4.98)	13.68 (3.34)	14.10 (3.84)	10.46 (3.50)	12.39 (4.17)	13.78 (1.33)	0.223	0.002
Glucose, mg/dL	86.63 (0.67)	86.85 (0.80)	86.51 (0.84)	87.21 (0.87)	88.05 (0.66)	88.45 (0.92)	89.80 (1.32)	89.37 (0.95)	87.11 (0.88)	89.35 (0.70)	87.89 (0.28)	0.032	<0.01
Prediabetes, %	4.23 (2.61)	2.56 (2.34)	3.92 (2.24)	0.55 (0.55)	4.59 (2.51)	4.04 (2.98)	8.56 (4.03)	9.84 (4.01)	2.65 (1.82)	4.22 (2.60)	4.44 (0.86)	0.372	0.275
AST, IU/L	16.41 (0.47)	15.86 (0.27)	15.93 (0.54)	17.49 (1.11)	15.33 (0.40)	15.80 (0.51)	16.82 (0.59)	16.39 (1.00)	17.05 (0.61)	17.36 (1.14)	16.44 (0.23)	0.206	0.207
ALT, IU/L	11.84 (0.56)	10.97 (0.49)	12.24 (1.17)	13.91 (2.09)	11.41 (0.51)	12.15 (1.08)	13.97 (1.44)	13.66 (2.30)	14.10 (1.23)	14.64 (3.62)	12.87 (0.54)	0.226	0.137
NAFLD, %	10.19 (2.77)	12.20 (3.63)	15.55 (4.15)	14.17 (4.58)	21.98 (4.75)	23.02 (5.87)	13.03 (3.65)	21.26 (4.98)	13.02 (3.87)	22.59 (5.10)	7.18 (1.01)	0.251	0.049

Continuous variables are presented as the mean (standard error) and categorical data as the percentage (standard error) *with Italic.* For calculating *p* value, analysis of variance was used to compare the mean values of the continuous variables and the Rao–Scott Chi-squared test was used to compare categorical variables. Linear trend analysis was performed using coefficients of orthogonal polynomials to calculate p for trend.
BMI: body mass index; SDS: standard deviation score; WC: waist circumference; AST: aspartate aminotransferase; ALT: alanine aminotransferase; NAFLD: non-alcoholic fatty liver disease.

Table S4. Trends of prediabetes of the subjects according to sex and BMI classification

Variable	2009	2010	2011	2012	2013	2014	2015	2016	2017	2018	*Total*	*P*	*P* for trend
**Male**													
Normal	n=411	n=324	n=296	n=276	n=276	n=204	n=216	n=228	n=211	n=209	n=2651		
WC, cm	68.67 (0.41)	67.86 (0.61)	67.17 (0.46)	67.49 (0.43)	67.54 (0.48)	68.52 (0.58)	70.50 (0.54)	68.14 (0.53)	68.75 (0.53)	69.15 (0.54)	68.31 (0.16)	<0.01	<0.01
Abdominal obesity, %	0.62 (0.61)	0.20 (0.20)	0.00 (0.00)	0.00 (0.00)	0.00 (0.00)	0.00 (0.00)	0.38 (0.38)	0.00 (0.00)	0.00 (0.00)	0.27 (0.27)	0.15 (0.09)	<0.01	0.506
Glucose, mg/dL	89.39 (0.34)	88.76 (0.43)	88.79 (0.48)	88.76 (0.43)	90.96 (0.50)	92.62 (0.62)	91.55 (0.43)	91.75 (0.47)	92.61 (0.53)	92.40 (0.56)	90.58 (0.16)	<0.01	<0.01
Prediabetes, %	4.64 (1.15)	4.12 (1.47)	6.90 (1.89)	3.39 (1.08)	8.35 (1.92)	15.12 (2.65)	9.44 (2.06)	10.68 (2.33)	15.51 (3.04)	13.33 (2.47)	8.74 (0.65)	<0.01	<0.01
AST, IU/L	19.04 (0.28)	19.32 (0.39)	19.13 (0.33)	20.03 (0.56)	18.78 (0.31)	20.11 (0.54)	20.24 (0.51)	19.73 (0.37)	21.37 (1.10)	20.57 (0.52)	19.78 (0.17)	0.013	<0.01
ALT, IU/L	14.24 (0.44)	13.95 (0.66)	13.40 (0.48)	13.91 (0.55)	13.71 (0.44)	15.58 (0.93)	14.73 (0.76)	14.32 (0.60)	17.95 (2.89)	15.90 (1.01)	14.69 (0.34)	0.248	0.023
NAFLD, %	4.58 (1.19)	3.62 (1.39)	4.20 (1.38)	6.19 (2.03)	4.44 (1.25)	9.64 (2.36)	5.70 (1.75)	10.59 (2.71)	7.35 (2.07)	7.06 (1.92)	6.17 (0.58)	0.083	<0.01
Overweight	n=51	n=50	n=28	n=32	n=39	n=22	n=35	n=32	n=22	n=25	n=336		
WC, cm	81.02 (0.68)	80.23 (0.84)	80.35 (1.43)	82.99 (1.11)	78.56 (1.56)	80.70 (0.92)	83.13 (0.84)	84.43 (0.86)	80.83 (1.29)	82.42 (1.44)	81.44 (0.38)	<0.01	0.040
Abdominal obesity, %	12.36 (4.79)	3.14 (1.95)	9.28 (5.59)	11.51 (6.93)	7.93 (4.17)	0.00 (0.00)	27.80 (7.68)	20.40 (7.58)	13.44 (8.04)	15.45 (10.76)	11.91 (1.98)	0.047	0.064
Glucose, mg/dL	90.64 (1.25)	91.76 (1.34)	89.59 (1.45)	88.68 (1.72)	91.88 (1.47)	94.61 (1.44)	94.14 (1.41)	92.61 (1.39)	95.15 (0.93)	90.10 (1.35)	91.72 (0.48)	<0.01	0.015
Prediabetes, %	14.05 (5.53)	7.43 (3.81)	13.07 (7.84)	3.96 (2.55)	12.16 (5.52)	15.19 (8.69)	16.04 (6.72)	22.00 (8.04)	18.69 (9.04)	5.31 (3.76)	12.50 (1.98)	0.493	0.343
AST, IU/L	21.78 (1.49)	23.12 (1.77)	25.42 (2.66)	22.40 (2.62)	22.57 (1.52)	19.83 (1.56)	22.15 (2.01)	19.89 (1.09)	20.36 (2.79)	25.86 (2.22)	22.33 (0.64)	0.276	0.630
ALT, IU/L	26.43 (4.19)	27.76 (4.36)	33.25 (7.14)	29.77 (8.53)	27.27 (3.76)	19.26 (2.72)	25.24 (4.89)	21.76 (2.39)	20.91 (6.38)	33.70 (9.05)	26.68 (1.78)	0.403	0.519
NAFLD, %	30.13 (7.93)	25.84 (7.20)	36.88 (11.35)	27.43 (9.61)	31.81 (9.04)	10.33 (5.94)	26.17 (7.25)	27.90 (9.24)	4.43 (4.37)	40.94 (12.48)	27.09 (2.90)	0.330	0.610
Obesity	n=39	n=45	n=41	n=32	n=32	n=26	n=42	n=42	n=26	n=35	n=360		
WC, cm	88.65 (1.15)	88.24 (1.31)	91.68 (1.38)	89.42 (0.96)	93.40 (1.88)	93.02 (1.81)	91.61 (1.59)	90.57 (1.50)	93.87 (1.35)	93.31 (1.58)	91.42 (0.49)	0.013	<0.01
Abdominal obesity, %	49.15 (9.14)	62.01 (8.33)	72.92 (8.73)	61.30 (8.23)	73.60 (8.46)	81.19 (8.60)	77.64 (6.29)	73.66 (7.21)	86.93 (7.18)	84.68 (7.14)	72.89 (2.60)	0.091	<0.01
Glucose, mg/dL	91.51 (1.13)	90.77 (1.23)	89.78 (0.88)	89.99 (1.70)	91.97 (1.11)	94.72 (1.23)	93.45 (0.92)	93.55 (1.26)	94.73 (1.08)	92.98 (1.24)	92.26 (0.42)	<0.01	<0.01
Prediabetes, %	16.22 (7.05)	10.15 (4.90)	0.00 (0.00)	4.35 (3.47)	14.62 (6.95)	20.27 (8.62)	11.55 (5.28)	12.71 (5.60)	19.61 (8.60)	12.92 (6.23)	11.44 (1.88)	0.155	0.123
AST, IU/L	24.60 (2.17)	24.32 (1.75)	27.93 (2.59)	30.26 (5.82)	27.12 (2.55)	22.77 (2.64)	30.98 (7.80)	22.97 (1.80)	24.33 (1.98)	27.04 (1.93)	26.45 (1.25)	0.658	0.845
ALT, IU/L	32.04 (5.17)	33.64 (3.79)	46.44 (9.11)	33.98 (6.94)	40.44 (4.98)	31.02 (4.92)	43.83 (14.36)	30.65 (5.33)	35.29 (5.05)	42.67 (4.92)	37.52 (2.50)	0.607	0.720
NAFLD, %	43.31 (9.20)	47.27 (8.49)	56.30 (9.22)	45.00 (10.45)	63.26 (9.13)	45.91 (12.53)	50.08 (8.32)	33.58 (8.79)	56.01 (11.04)	70.77 (8.57)	51.17 (3.12)	0.261	0.388
**Female**													
Normal	n=388	n=295	n=267	n=261	n=274	n=169	n=187	n=222	n=204	n=181	n=2448		
WC, cm	64.37 (0.39)	64.68 (0.41)	64.44 (0.50)	64.43 (0.40)	65.04 (0.44)	65.53 (0.57)	66.29 (0.40)	65.28 (0.48)	64.29 (0.53)	64.18 (0.47)	64.82 (0.14)	<0.01	0.373
Abdominal obesity, %	0.79 (0.40)	1.03 (0.57)	1.79 (0.92)	0.10 (0.10)	2.57 (1.27)	2.41 (1.34)	0.81 (0.57)	1.75 (0.84)	1.29 (1.28)	0.68 (0.68)	1.32 (0.28)	0.467	0.774
Glucose, mg/dL	87.94 (0.34)	87.68 (0.43)	87.75 (0.51)	88.98 (0.51)	89.48 (0.48)	89.40 (0.48)	89.97 (0.50)	90.34 (0.49)	89.17 (0.51)	90.29 (0.51)	89.03 (0.15)	<0.01	<0.01
Prediabetes, %	3.71 (1.10)	2.69 (0.86)	5.47 (1.78)	1.76 (0.82)	6.88 (1.66)	4.51 (1.51)	5.32 (1.74)	7.32 (1.90)	5.19 (1.50)	6.35 (1.86)	4.87 (0.48)	0.131	0.026
AST, IU/L	17.62 (0.28)	17.18 (0.31)	16.96 (0.29)	16.68 (0.27)	16.59 (0.24)	17.05 (0.37)	17.63 (0.35)	17.08 (0.51)	17.44 (0.30)	18.22 (0.47)	17.23 (0.11)	0.018	0.074
ALT, IU/L	11.28 (0.28)	10.87 (0.32)	10.53 (0.26)	10.77 (0.34)	11.22 (0.31)	10.81 (0.51)	11.19 (0.44)	11.35 (1.07)	10.80 (0.34)	10.98 (0.48)	10.98 (0.15)	0.750	0.817
NAFLD, %	4.57 (1.30)	2.17 (0.86)	1.62 (1.04)	1.88 (0.93)	2.27 (0.94)	2.16 (1.27)	4.70 (1.56)	4.64 (1.76)	2.71 (1.06)	2.04 (0.98)	2.87 (0.38)	0.363	0.899
Overweight	n=48	n=31	n=34	n=21	n=29	n=23	n=21	n=18	n=28	n=19	n=272		
WC, cm	74.48 (0.83)	75.93 (1.31)	73.96 (1.18)	76.31 (0.77)	73.98 (0.68)	75.93 (0.55)	74.77 (0.90)	78.19 (1.36)	73.80 (0.72)	75.25 (1.40)	75.14 (0.33)	0.036	0.556
Abdominal obesity, %	21.91 (6.86)	48.41 (10.38)	26.52 (9.56)	17.08 (9.34)	10.72 (5.97)	23.48 (9.61)	21.59 (8.60)	48.63 (11.58)	7.59 (5.39)	21.82 (11.51)	24.12 (3.00)	0.028	0.300
Glucose, mg/dL	87.05 (0.80)	90.14 (1.63)	89.02 (1.57)	89.58 (1.21)	92.12 (1.46)	88.68 (1.51)	90.24 (1.16)	91.14 (1.31)	87.99 (1.67)	93.07 (1.58)	89.68 (0.47)	0.013	0.082
Prediabetes, %	0.91 (0.92)	11.06 (5.17)	9.73 (6.52)	2.40 (2.44)	10.88 (6.05)	8.43 (5.44)	0.00 (0.00)	0.00 (0.00)	6.61 (4.62)	8.54 (5.90)	6.00 (1.50)	0.198	0.904
AST, IU/L	16.56 (0.66)	17.14 (0.77)	16.34 (0.62)	23.72 (3.90)	17.53 (0.92)	15.32 (0.60)	16.38 (0.84)	17.45 (0.92)	16.82 (1.03)	23.19 (5.24)	17.89 (0.66)	0.207	0.518

ALT, IU/L	12.56 (0.64)	11.26 (0.51)	12.15 (0.79)	24.01 (7.76)	14.57 (1.94)	10.79 (0.89)	13.56 (1.75)	13.66 (1.14)	13.34 (1.20)	31.92 (19.11)	15.32 (1.79)	0.118	0.382
NAFLD, %	5.46 (2.70)	1.44 (1.43)	1.64 (1.62)	24.40 (11.92)	7.70 (4.46)	0.77 (0.79)	9.63 (8.97)	11.72 (8.13)	8.51 (5.52)	12.39 (8.42)	7.99 (2.01)	0.047	0.269
Obesity	n=28	n=26	n=33	n=21	n=22	n=22	n=34	n=27	n=26	n=21	n=260		
WC, cm	81.96 (1.34)	82.90 (1.68)	82.74 (1.20)	81.02 (0.82)	80.72 (1.82)	81.56 (1.19)	86.75 (1.79)	83.88 (1.11)	84.18 (2.23)	83.06 (1.63)	83.17 (0.53)	0.162	0.115
Abdominal obesity, %	65.06 (10.71)	71.05 (12.31)	77.00 (7.80)	91.90 (4.71)	65.64 (10.95)	71.57 (10.54)	87.28 (5.03)	88.24 (5.64)	74.86 (9.44)	61.48 (11.94)	76.26 (3.00)	0.207	0.819
Glucose, mg/dL	8.80 (5.29)	14.75 (8.33)	12.17 (6.39)	9.46 (6.15)	0.00 (0.00)	5.79 (5.65)	24.77 (8.61)	26.63 (9.22)	0.00 (0.00)	22.34 (10.22)	91.13 (0.68)	0.014	0.338
Prediabetes, %	89.76 (1.95)	91.14 (2.68)	90.55 (1.68)	86.36 (2.18)	88.55 (1.29)	92.15 (1.62)	94.35 (2.66)	93.32 (1.82)	89.13 (1.22)	93.13 (2.05)	2.38 (1.19)	0.112	0.082
AST, IU/L	18.19 (0.67)	17.00 (0.60)	18.34 (1.39)	15.83 (0.98)	15.59 (1.03)	16.87 (1.01)	20.05 (1.86)	18.71 (1.80)	22.17 (2.24)	19.75 (2.34)	18.53 (0.55)	0.059	0.023
ALT, IU/L	19.97 (2.10)	14.42 (1.28)	20.14 (3.38)	14.94 (2.44)	15.44 (1.31)	16.53 (1.67)	21.07 (3.24)	20.71 (3.54)	27.88 (5.13)	21.28 (4.86)	19.72 (1.14)	0.049	0.046
NAFLD, %	31.62 (10.98)	7.51 (6.06)	28.82 (7.79)	16.35 (9.86)	21.52 (9.89)	13.31 (7.40)	25.29 (9.26)	30.52 (10.28)	51.73 (10.44)	30.23 (10.49)	26.60 (3.16)	0.098	0.077

Continuous variables are presented as the mean (standard error) and categorical data as the percentage (standard error) *with Italic.* For calculating *p* value, analysis of variance was used to compare the mean values of the continuous variables and the Rao–Scott Chi-squared test was used to compare categorical variables. Linear trend analysis was performed using coefficients of orthogonal polynomials to calculate p for trend.
BMI: body mass index; WC: waist circumference; AST: aspartate aminotransferase; ALT: alanine aminotransferase; NAFLD: non-alcoholic fatty liver disease.
